# CD8^+^ T cell-based cancer immunotherapy

**DOI:** 10.1186/s12967-024-05134-6

**Published:** 2024-04-29

**Authors:** Yanxia Chen, Dingning Yu, Hui Qian, Yinghong Shi, Zhimin Tao

**Affiliations:** 1 Jiangsu Key Laboratory of Medical Science and Laboratory Medicine, Department of Laboratory Medicine, School of Medicine, Jiangsu University, Zhenjiang, Jiangsu 212013 China; 2 Department of Laboratory Medicine, Shaoxing People’s Hospital, Shaoxing, Zhejiang 312000 China; 3 Zhenjiang Key Laboratory of High Technology Research on Exosomes Foundation and Transformation Application, School of Medicine, Jiangsu University, Zhenjiang, Jiangsu 212013 China; 4 Department of Emergency Medicine, The Affiliated Hospital of Jiangsu University, Zhenjiang, Jiangsu 212001 China

**Keywords:** CD8^+^ T cell, Cancer therapy, CAR-T, Checkpoint inhibitor, Solid tumor

## Abstract

The immune system in humans is a defense department against both exogenous and endogenous hazards, where CD8^+^ T cells play a crucial role in opposing pathological threats. Various immunotherapies based on CD8^+^ T cells have emerged in recent decades, showing their promising results in treating intractable diseases. However, in the fight against the constantly changing and evolving cancers, the formation and function of CD8^+^ T cells can be challenged by tumors that might train a group of accomplices to resist the T cell killing. As cancer therapy stepped into the era of immunotherapy, understanding the physiological role of CD8^+^ T cells, studying the machinery of tumor immune escape, and thereby formulating different therapeutic strategies become the imperative missions for clinical and translational researchers to fulfill. After brief basics of CD8^+^ T cell-based biology is covered, this review delineates the mechanisms of tumor immune escape and discusses different cancer immunotherapy regimens with their own advantages and setbacks, embracing challenges and perspectives in near future.

## Introduction

The basics of cancer immunotherapy is to initiate and optimize the key procedures in the innate or acquired immune system, including but not limited to, surveillance, identification, and elimination of tumors [1]. The ultimate executors of acquired cellular immunity, i.e., CD8^+^ T cells, are exceptionally destructive when encountering tumors [2]. The advent of novel cancer treatments, such as immune checkpoint blockers, neoantigen vaccines, chimeric antigen receptor T (CAR-T) cell therapy and T-cell receptor T (TCR-T) cell therapy, points to that CD8^+^ T cell-based immunotherapy has gained dramatic breakthroughs. Recently, autologous cell therapy using tumor-infiltrating lymphocytes (TILs) has been approved by the United States (US) Food and Drug Administration (FDA) to treat patients with unresectable or metastatic melanoma, further bolstering the already rapid development of novel CD8^+^ T cell-involved therapy [3].

Under normal circumstances, the immune system monitors the aberrant activities in the body and identifies and removes the suspicious components (e.g., tumor cells), thereby preventing tumor initiation. However, in patients suffering from cancers, the fulfillment of immune surveillance can go awry. A variety of immune escape strategies might be adopted by tumors to counteract the host’s monitoring, thus leading to uncontrolled tumor growth [4, 5]. Under these circumstances, the effective functions of CD8^+^ T cells can be remarkably disturbed. Therefore, exploration of constantly changing immune escape mechanisms and development of the emerging CD8^+^ T cell-based immunotherapy strategies make the driving seats behind the wheel of cancer research (1).

**Table 1:** Clinical trials of CD8^+^ T cell-based cancer immunotherapy

Types of Immunotherapy	Target	Drugs/Biologics	Types of cancer (s)	NCT number
Immune checkpoint blocker therapy	Anti-PD 1/PD-L1	Nivolumab (Opdivo)	Glioblastoma	NCT02550249, NCT03430791, NCT02529072
		Pembrolizumab	Hodgkin Lymphoma	NCT02453594, NCT02875067
		Tislelizumab (BGB-A317)	Lung Cancer	NCT03745222, NCT04005716
		Cemiplimab (REGN2810)	Non-Small Cell Lung Cancer	NCT03367819, NCT03515629
		Avelumab	Gastric Cancer	NCT02625623, NCT02625610
		Durvalumab	Squamous Cell Carcinoma	NCT03144738, NCT02262741
		Atezolizumab	Cutaneous Melanoma	NCT04020809
			Colorectal Carcinoma	NCT02788279
			Bladder Cancer	NCT03359239, NCT02951767
			Metastatic Pancreatic Cancer	NCT04820179
	Anti-CTLA-4	Ipilimumab	Prostate Cancer	NCT02113657, NCT01804465, NCT00064129
		Tremelimumab	Melanoma	NCT02535078, NCT00378482
Cancer vaccine	Peptide vaccines	HER2/Neu Peptide Vaccine	Breast Cancer Colorectal Cancer	NCT00854789, NCT03391232, NCT00005632 NCT00091286
		Polypepi1018 CRC vaccine	Colorectal Carcinoma	NCT05130060
		MUC1-KLH vaccine	Prostate Cancer	NCT00005632
	Dendritic Cell vaccine	NY-ESO-1 peptide-pulsed autologous dendritic cell vaccine	Solid Neoplasm Melanoma (Skin)	NCT02775292 NCT00798629
		Autologous dendritic cell vaccine	Breast Cancer	NCT04879888, NCT00019084
		Tumor blood vessel antigen peptide-pulsed alpha-type-1 polarized dendritic	Metastatic Breast Cancer	NCT02479230
		cell vaccine		
	DNA vaccine	Mammaglobin-A DNA vaccine	Metastatic Breast Cancer	NCT00807781
		Personalized polyepitope DNA vaccine	Triple Negative Breast Cancer	NCT02348320
	RNA vaccine	CV9103	Hormonal Refractory Prostate Cancer	NCT00831467, NCT00906243
		RBL001/RBL002	Melanoma	NCT01684241
	Neoantigen vaccines	Personalized vaccine	Melanoma	NCT01970358, NCT03480152
		NEO-PV-01	Non-Small Cell Lung Cancer	NCT03380871
Cell therapy	Adoptive Cell Transfer Therapy	Therapeutic autologous lymphocytes	Brain and Central Nervous System Tumors	NCT00730613, NCT00331526, NCT00003158
		Autologous cytokine-induced killer cells	Hepatocellular Carcinoma	NCT03124498
	CAR-T cells	Anti-meso CAR-T cells	Mesothelioma	NCT01355965, NCT02159716
		Anti-CD19 CAR-T cells	Lymphoblastic Leukemia	NCT02975687, NCT03076437
		Anti-CEA CAR-T cells	Pancreatic Cancer	NCT02850536
		Anti-GPC3 CAR-T cells	Hepatocellular Carcinoma	NCT02395250
		Anti-CD19/22 CAR-T cells	Acute Lymphoblastic Leukemia	NCT05418088, NCT05674175
		Anti-BCMA CAR-T cells	Multiple Myeloma	NCT02658929, NCT03455972
		Anti-EGFRVIII CAR-T cells	Glioblastoma	NCT01454596
		Anti-GD2 CAR-T cells	Melanoma	NCT02107963
		Anti-CD133 CAR-T cells	Colorectal Cancer	NCT02541370
	TCR-T cells	Autologous WT1 TCR transduced T cells	Myeloid Leukemia	NCT02550535, NCT02770820
			Non-Small Cell Lung Cancer	NCT02408016
		Anti-NY-ESO-1 engineered T cells	Soft-Tissue Sarcomas	NCT05296564
		Anti-P53 T-cell receptor transduced lymphocytes	Kidney Cancer	NCT00704938
		NYESO-1 T cells	Melanoma	NCT02062359, NCT00518206, NCT01967823
Nanomedicine treatment	Nano drug	Magnetic nanoparticle	Prostate Cancer	NCT02033447, NCT00147238
		Carbon nanoparticles	Thyroid Cancer	NCT02724176, NCT06048367
		Docetaxel nanoparticles (BIND-014)	Prostate Cancer Non-Small Cell Lung Cancer	NCT01812746 NCT01792479
		Nab-Paclitaxel (Abraxane)	Biliary Cancers	NCT02392637
		Ferumoxytol (Feraheme)	Bladder Cancer	NCT02141490
		BP-C1	Pancreatic Cancer	NCT03627390
		Paclitaxel (Genexol®)	Breast Cancer	NCT00876486

Nevertheless, albeit immunotherapy demonstrated tremendous success in oncological treatment, a relatively small fraction of patients with cancers, especially solid tumors, responded to a diversity of immunotherapies [6, 7]. Concurrently, incidents of adverse events and cancer recurrence following immunotherapy still exist [8–10]. Therefore, in this review, we summarize the recent advances on CD8^+^ T cell-based cancer therapy, aiming to introduce the basics of CD8^+^ T cells through their generation, activation, function, and destination, together with new research findings, and to delineate the machinery regarding the immune escape of tumors by deceiving CD8^+^ T cells and immunotherapy regimens based on activation CD8^+^ T cells, all mingled with challenges and progresses.

## The life cycle of CD8^+^ T cells

**Fig. 1:**
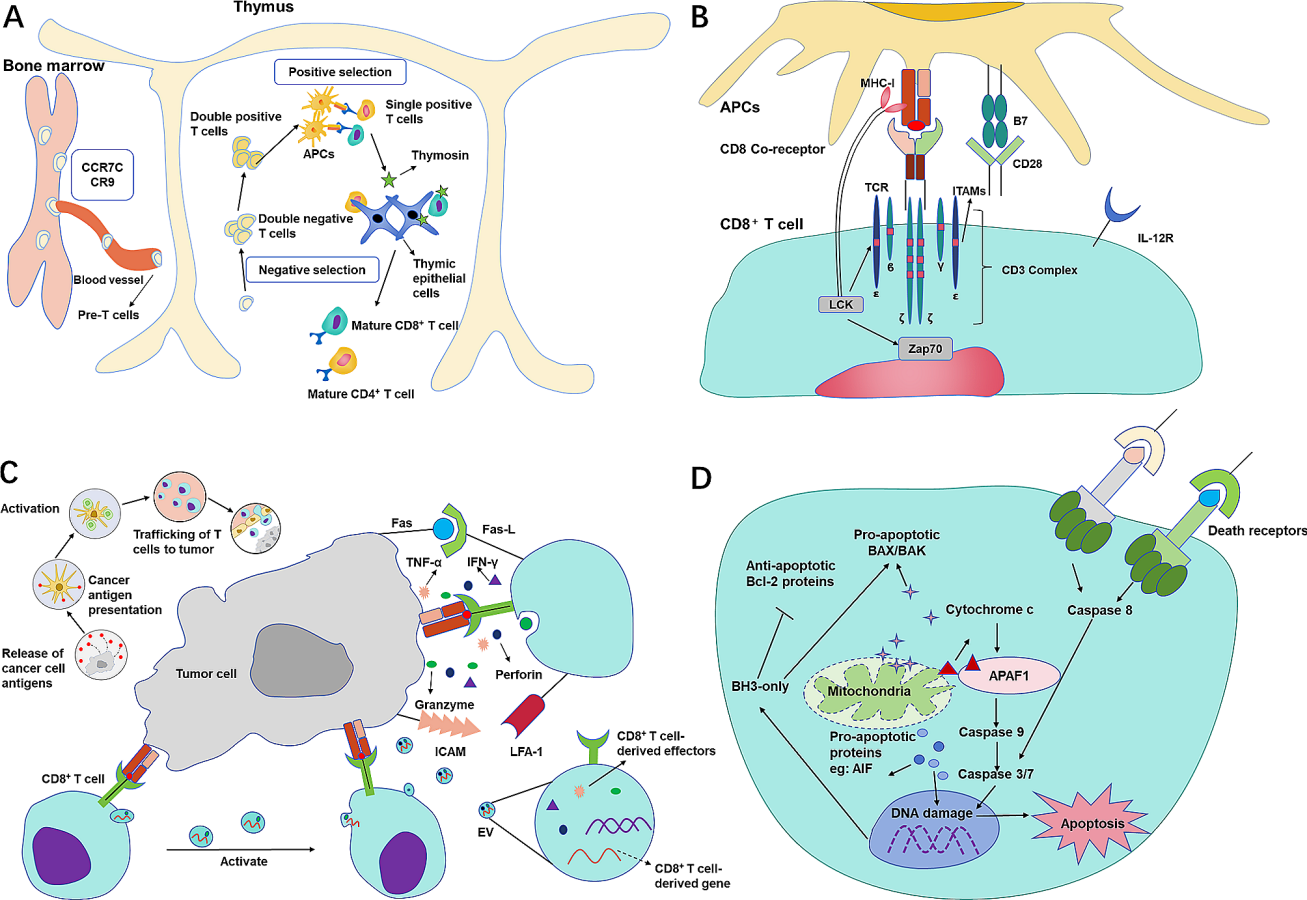
The life cycle of CD8^+^ T cells. (**A**) Birth of mature CD8^+^ T cells. Red bone marrow produces pre-T cells, which are recruited to the thymus under the action of chemokines. Subsequently, T cells undergo positive and negative selection to become mature CD4^+^T or CD8^+^T cells, assisted by antigen-presenting cells (APCs), thymic epithelial cells and thymic factors. (**B**) Activation of CD8^+^ T cells. The activation of CD8^+^ T cells requires the assistance of three signals, namely the precursor signal triggered by the binding of TCR and MHC-I, the ligand mediated co-stimulatory signal and the supplementary signal produced by cytokines. ITAM, immunoreceptor tyrosine-based activation motif; LCK, lymphocyte-specific protein tyrosine kinase. (**C**) Anti-tumor effect of CD8^+^ T cells. CD8^+^ T cells can form immune synapses with target cells after activation. Subsequently, CD8^+^ T cells release granzymes, perforin, and cytokines to destroy tumor cells. Meanwhile, CD8^+^ T cells express the death receptor Fas-L and induce apoptosis in Fas expressing tumor cells. In addition, activated CD8^+^ T cells may kill target cells through the delivery of EVs. Tumor antigens, once released, are presented by DCs and T cells are activated to infiltrate tumors, recognizing and dismantling tumor cells. LFA-1, lymphocyte function-associated antigen 1; ICAM, intercellular cell adhesion molecule. (**D**) The fate of CD8^+^ T cells. CD8^+^ T cells expressing death ligand receptors can induce activated cell death of their own or adjacent CD8^+^ T lymphocytes. Meanwhile, apoptosis related gene 2 can accelerate the CD8^+^ T cell death by cleaving the differentiation protein of myeloid leukemia cells. The expression of pro-apoptotic proteins BAX and BAK enhances mitochondrial membrane permeability, leading to the activation of cytochrome c and its binding to apoptotic protease activating factor-1, further leading to caspase 8/-3 dependent cell death

Red bone marrow produces the precursor T cells, which are recruited to the thymus under the action of chemokines [11]. In the thymus, mature CD8^+^ or CD4^+^ T cells are transported to the peripheral blood after positive and negative selection with assistance of antigen-presenting cells (APCs), thymic epithelial cells and thymus factors (Fig. 1A) [12]. The complete activation of antigen-specific CD8^+^ T cells requires the synergy combining three types of signals, including the pioneer signals initiated by TCR binding to peptide-bound major histocompatibility complex (MHC), the ligand-mediated costimulatory signal, and the supplementary signals produced by cytokines [13, 14]. Eventually, the above signals initiate a cascade of effector proteins that accelerate T cell activation (Fig. 1B).

Antigen-specific CD8^+^ T cells are activated after recognizing tumor-associated antigens presented by MHC class I (MHC I) molecules, and an immune synapse is formed between the target tumor cells and CD8^+^ T cells of high functional and structural avidity that preferentially reside in tumors [15]. A lately study also revealed that neonatal CD8^+^ T cells could undergo a bystander activation in response to innate cytokines without cognate TCR stimulation [16]. But whether this TCR-independent activation take place in the context of tumor killing remains unknown. Consequently, CD8^+^ T cells release granzymes, perforins and cytokines through the immune synapse in a high density to destroy tumor cells, and on many occasions this cytotoxicity could be delivered via T cell-derived extracellular vesicles (EVs) [17, 18]. In parallel, CD8^+^ T cells can express the death receptor Fas-L and induce apoptosis in Fas-expressing tumor cells [19], or kill target cells through other destructive pathways, such as pyroptosis [20, 21] or ferroptosis [22] (Fig. 1C).

As activated CD8^+^ T cells cannot proliferate or survive indefinitely, their fate is rigorously manipulated by several immune balance mechanisms. Those cells expressing both death receptors and ligands can induce activated cell death of self or adjacent CD8^+^ T cells [23]. At the same time, CD8^+^ T cells can maintain their own homeostasis in a death receptor-independent way. For example, apoptosis-related gene 2 can accelerate the death of CD8^+^ T cells by lysing myeloid leukemia cell differentiation proteins [24]. Pro-apoptotic proteins BAX and BAK are expressed, and the permeability of mitochondrial membrane is enhanced, resulting in the activation of cytochrome c and its binding to apoptotic peptidase activator 1 for further caspase-8/-3-dependent cell death [25]. Additionally, CD8^+^ T cells can be eliminated through immune checkpoint inhibitory signaling [26], transforming growth factor b (TGF-b) signaling [27] or/and autophagy [28] (Fig. 1D).

## Cancer immune escape: in a close relation to CD8^+^ T cells

### Cancer cell

#### Lack of immunogenicity

Activation of specific CD8^+^ T cells requires the presence of recognizable tumor antigens, while the lack of immunogenicity for certain antigens may be the reason for the failure of T cell immune response [29]. Cancer cells can downregulate or neutralize self-antigens to lower immunogenicity, so deceiving the immune system [30]. In the early stage of tumor progression, most MHC I molecules on the surface of tumor cells act as a medium for CD8^+^ T cells to recognize, so to kill them [31]. Unfortunately, a variety of tumor cells downregulate human leukocyte antigen (HLA) genes to reduce MHC I molecules so that the decrease of tumor antigen presentation tricks the CD8^+^ T cells into skipping their killing [32]. As a result, CD8^+^ T cells eliminate most of the MHC I-positive tumor cells, while MHC I-negative tumor cells survive and grow [33]. Additionally, genes related to antigen processing such as ER-resident aminopeptidase are frequently downregulated in tumor microenvironment (TME) [34].

Many types of tumor cells can alter their surface glycosylation to evade the immune response of T cells. Those can express sialic acid-carrying glycans to cover the cell membrane, so preventing the maturation of dendritic cells (DCs) in the TME and impairing their antigen-presenting functions [35]. The sialic acid blockers can thus reduce the sialylation, restore the tumor immunogenicity, and promote the aggregation and cytotoxicity of CD8^+^ T cells to clear tumors [36]. In addition, CD39 is a rate-limiting enzyme in the process of ATP conversion to adenosine, as high expression of CD39 on the tumors surface represses the maintenance of extracellular ATP inflammatory signals, leading to the formation of non-inflammatory tumors and loss of immune response [37].

#### Secretion of immunosuppressive factors and enzymes

Tumor cells can upregulate the expression of multiple immunosuppressive substances to help their evasion [38]. For example, together with the ATF1 transcription factor, SMAD protein activated by TGF-β can bind and inhibit the promoter genes of the granzyme B and interferon γ (IFN-γ), thereby restraining the cytotoxic function of CD8^+^ T cells [39]. Concomitantly, TGF-β can diminish expression of chemokine receptor CXC motif 3 (CXCR3) that is primarily expressed on activated CD8^+^ T cells, by increasing SMAD protein binding to CXCR3 promoter, so restricting their trafficking into tumors [40]. Similarly, the secretion of IL-37b in melanoma cells downregulates the co-stimulatory molecules on DCs, then imposing severe damage on CD8^+^ T cell activation [41]. However, an atypical example can be IL-10, an anti-inflammatory cytokine, which has the dual role of inhibiting [42] and promoting tumors [43, 44]. IL-10 can suppress myeloid and chronic inflammatory T cell responses and expand tumor specific CD8^+^ T cells. On the contrary, IL-10 also induces IFN-γ and cytotoxic mediators in antigen-activated T cells [45]. Thus, IL-10 plays a crucial role in the shift from activation to exhaustion in T cells.

Indoleamine 2, 3-dioxygenase 1 (IDO1) is highly expressed on many cancers, being a pivotal enzyme in the pathway of tryptophan metabolism [46]. It has been well established that tryptophan is essential for T cell proliferation and activation [47]. Tryptophan metabolites own effective T-cell-inhibiting capacities and can enhance the differentiation and immunosuppression ability of regulatory T cells (Tregs), thus promoting the progression of several cancers, including colon cancer and pancreatic adenocarcinoma [48, 49]. A study further demonstrated that IDO1 could inhibit the response of CD8^+^ T cells and promote the tumor growth in subcutaneous colon cancer models, highlighting the role of IDO1 in tumor immune escape [50].

#### Expression of immunosuppressive coreceptors

Under the assistance of co-stimulation signals, CD8^+^ T cells can be further activated to accurately regulate the autoimmunity. These co-stimulatory signals deliver suppressing or promoting messages to T cells so that the presence of inhibitory receptors brings the negative regulation of T cell response to avoid damaging autoimmunity [51]. However, untamable tumor cells can frequently raise the expression of regulatory proteins on the cell surface, including cytotoxic T lymphocyte antigen 4 (CTLA-4), Fas-L, programmed cell death-ligand 1 (PD-L1), lymphocyte activation gene-3 (LAG-3) and T cell immunoglobulin and ITIM domain (TIGIT), so triggering T cell apoptosis [52]. For instance, gastric adenocarcinomas infiltrated by high density CD8^+^ T cells can express high levels of PD-L1 to avoid immune surveillance [53].

Understanding towards several tumor-related signaling pathways, such as Wnt/β-catenin, MAPK, EGFR, and STAT, has uncovered partial mechanisms involved in cancer immune escape [54]. Among them, Wnt/β-catenin signaling pathway has been proven a major route of tumor immunosuppression in colorectal cancer, breast cancer and melanoma [55]. β-catenin can promote the expression of transcription suppressor ATF3 to downregulate CCL4, so to impede the recruitment of DCs and CD8^+^ T cells into tumors [56]. In parallel, β-catenin promotes Tregs differentiation by stimulating tumor production of IDO1 and synergistically suppressing the immune response [57].

#### Release of EVs

Tumors can evade immune killing by secreting small EVs (traditionally known as exosomes), containing soluble factors, enzymes, immunoregulatory receptor ligands and RNAs, to interfere with the immune system [58]. Exosomes containing TGF-β released by tumor cells under hypoxic conditions promote the activation of Tregs [59]. Tregs co-cultured with exosomes derived from tumor cells also showed high expressions of Fas-L and CTLA-4, ably inhibiting the function of CD8^+^ T cells [60].

The arena of tumor exosomes is not limited to TME. For instance, exosomes secreted by melanoma cells are abundant in PD-L1, which can confront CD8^+^ T cells in the circulatory system and undermine their function. In addition, IFN-γ upregulates PD-L1 in exosomes [60]. Exosomes that originate from melanoma contain a variety of chemokines (CXCL1, CXCL10, CCL2, CCL5) to recruit immunosuppressive cells such as neutrophils [61]. After mRNAs in tumor-derived exosomes are transferred to T cells, the mRNA transcript is finally translated into immunosuppressive proteins, indicating that tumor-derived exosomes can induce immune tolerance through reprogramming [62]. Alternatively, CD39^+^ Tregs are continuously stimulated by CD73 in tumor-derived exosomes, causing adenosine upregulation [63, 64]. Thus, adenosine interacts with adenosine A2A receptor to rise cAMP level, therefore triggering CD8^+^ T cell anergy [65].

### Tumor suppressive environment

#### Immunosuppressive cell infiltration

##### Tregs

**Fig. 2:**
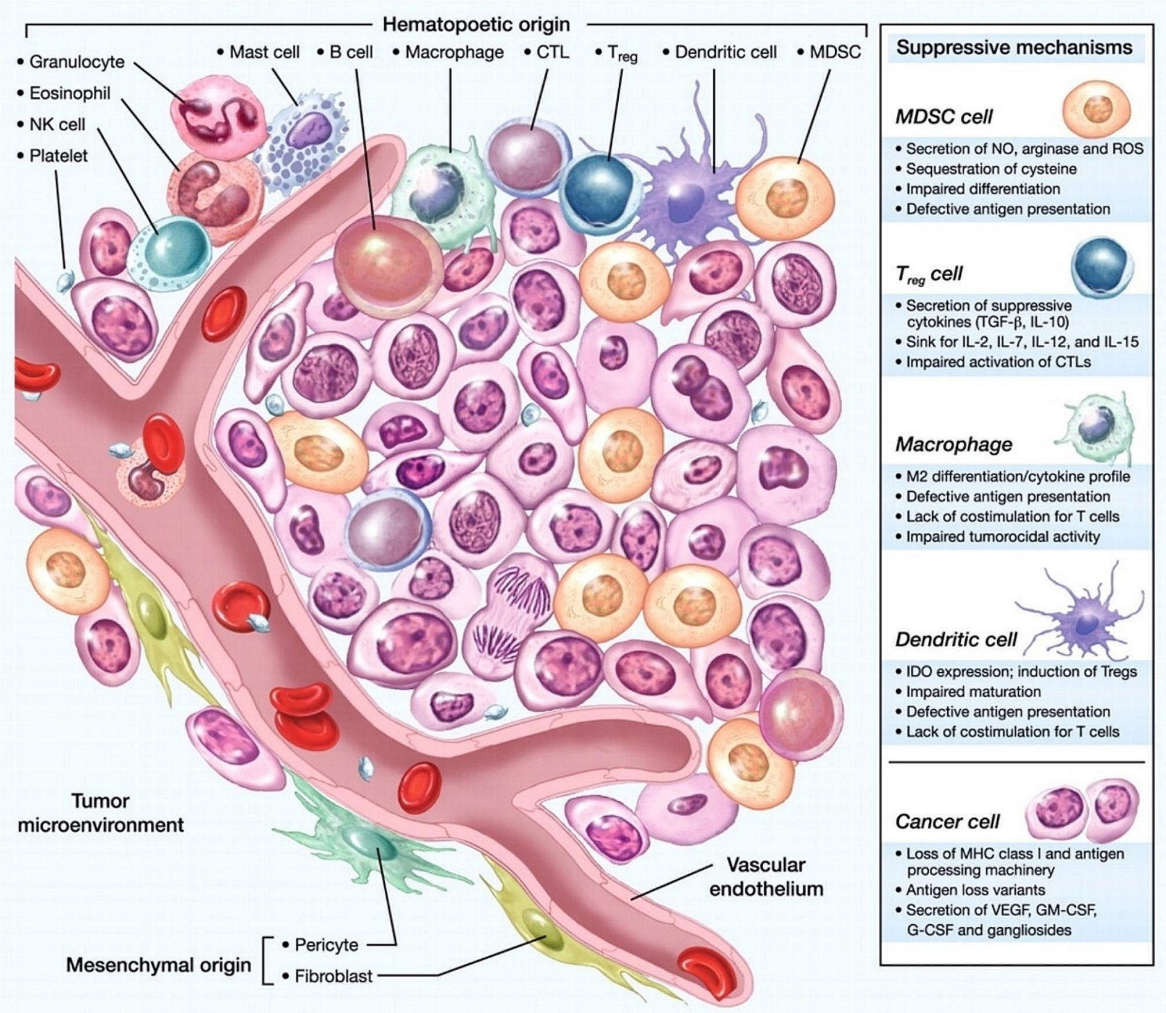
Intratumoral immune cells form a complex network that promotes tumor immune escape. TME hosts infiltrating immune cells, including MDSCs, DCs, mast cells, macrophages, neutrophils, lymphocytes, and natural killer cells with aberrant functions, which may work synergistically in a group to accomplice with tumors to evade immune surveillance and destruction, thereby promoting tumor growth. Reproduced with permission [156]

Tregs are the primary factors in the formation of immunosuppressive environments in tumors [66]. Myeloid-derived inhibitory cells and tumor cells upregulate the expression of CCL5, CCL22 and CCL28 to attract Tregs expressing CCR5, CCR10 or CCR4 to migrate toward tumors [67]. Tregs can express inhibitory receptors such as CTLA-4, T cell immunoglobulin and mucin-domain containing-3 (TIM-3), PD-1, GITR, LAG-3, BTLA and NRP-1, and secrete active substances such as IL-10, TGF-β, IL-2, IL-35, IDO1 and adenosine, respectively, through various mechanisms to inhibit the differentiation, activation and function of CD8^+^ T cells [68]. Tregs, an enhancer of immune suppression, inhibit the secretion of anti-inflammatory factors [69] (Fig. 2).

##### Tolerogenic DCs

Dendritic cells are professionals who present antigens to CD8^+^ T cells through MHC I molecules [70]. Mature DCs secrete IL-12 and IFN-α/-β as well as upregulate co-stimulating receptors such as CD80/86 to offer signals for CD8^+^ T cell differentiation and activation [71]. However, tumor cells in the TME can secrete inhibitory cytokines (IL-10, TGF, and RANKL) and decrease expression of costimulatory molecules to impede the maturation of DCs, converting it to a tolerance phenotype. The tolerogenic DCs produce IDO and IL-10, and express inducible co-stimulatory molecule ligand to stimulate the production of Tregs, finally inhibiting the immune response of CD8^+^ T cells [72].

##### TAMs

The exact role of tumor-associated macrophages (TAMs) in the tumor proximity depends on the macrophage phenotype conferred by TME [73]. Some TAMs play an active role in antigen presentation, while others, such as senescent TAMs, can suppress T-cell-mediated anti-tumor immune response, thus promoting tumor progression [74]. TAMs secrete immunosuppressive mediators such as IL-10 and TGF-β and downregulate receptors of Fas-L, PD-L1 and CD80/86, imposing negative impacts on CD8^+^ T cells, directly or indirectly [75]. It has been reported that TAMs can express CCL22, CCL17, and CCL18 to promote Tregs recruitment [76].

##### Neutrophils

Human neutrophils can be divided into two categories, namely N1 with anticancer effect and N2 with immunosuppressive effect [77]. N2 is an abnormal immature neutrophil or infiltrating mature neutrophils induced by TGF-β in TME [78]. Moreover, N2 neutrophils can induce the production of nitric oxide synthase, argininase 1 and reactive oxygen species, and prevent T cells from producing the active ingredient IFN-γ [79]. Besides, the high level of N2 neutrophil with expression of CCL2 and CCL17 could promote the aggregation of TAMs and Tregs and assist the maintenance of tumor immunosuppressive environment [80].

#### Other suppressive tactics

Cancer cells can evade immune surveillance while preserving immunogenicity and continue to stimulate the immune system, initiating a series of aberrant immune behaviors that further assist in tumor immune escape [81]. For instance, the constant stimulation of tumor antigens motivates T cells to work persistently, eventually reaching a state of exhaustion [64]. Canonically, the more severely depleted T cells are, the more inhibitory receptors are expressed on the cell surface, reducing the sensitivity to antigenic stimulation [82].

Solid malignancies are usually accompanied by anoxic acidic microenvironments [83]. Hypoxia-inducible factor-α (HIF-α) accumulates under these conditions and is associated with the initiation of multiple genes, especially key genes in angiogenesis and glycolytic pathway [84]. Cox-2/PGE2 pathway can enhance the activity of HIF2-α, activate the TGF/EGFR pathway to accelerate the nuclear transfer of HIF2-α, bind to the hypoxia binding region of vascular endothelial growth factor (VEGF) and cyclin D1 encoding gene promoter, thereby mediating the progression of lung cancer [85]. Hypoxic environment increases the expression of immunosuppressive receptor PD-L1 in tumor cells with HIF1-α present, while overexpressed PD-L1 interacts with programmed cell death protein-1 (PD-1) of CD8^+^ T cells to promote apoptosis of CD8^+^ T cells [86]. Furthermore, CD38 in hypoxic tumor environment takes partial responsibility for the formation of adenosine, while extracellular adenosine binds to adenosine receptors on CD8^+^ T cells, thus restraining CD8^+^ T cell activation and recruiting Tregs to further increase immune resistance in tumor cells [87].

Hypoxia-induced oncogene activation enhances glycolysis and lactic acid accumulation, leading to acidification in TME [84]. The pH-sensing protein mechanism allows tumors to survive in an acidic environment, while CD8^+^ effector and CD8^+^ memory T cells suffer from impaired function and shortened lifespan [88]. Thus, hypoxia-mediated extracellular acidification prevents T cells from expanding or performing their cytotoxic effects.

## Cancer immunotherapy involving CD8^+^ T cells

### Immune checkpoint therapy

Expressed on immune cells, immune checkpoints are a group of immunosuppressive molecules that can regulate the degree of immune activation and avoid autoimmune responses, so maintaining immune tolerance [89]. However, to change the fate of being eliminated, some tumor cells obviate such mechanism and deliver signal stimulation to immune cells, triggering T cell dysfunction and apoptosis [52]. Immunotherapy-based treatments, including immune checkpoint inhibitors (ICIs) typified by anti-PD-1/PD-L1 drugs, can re-activate immune cells by blocking immune checkpoints in cancer patients, so CD8^+^ T cytotoxicity against tumor cells can be restored [90].

In recent years, ICIs revolutionize the cancer treatment [91]. Research on monoclonal antibodies (mAbs) as inhibitors against the immune checkpoints that include PD-1, CTLA-4, TIGIT, LAG-3, and TIM-3, has undertaken a flurry of breakthroughs [92]. Among them, ipilimumab (anti-CTLA-4 mAb) is the first anti-cancer immunotherapy drug approved by the US FDA to treat unresectable or metastatic melanoma [93]. Since then, clinical development of antibody drugs based on immune checkpoint blockade has been upsurging. In 2015 and 2016, nivolumab (anti-PD-1 mAb) was respectively approved to treat metastatic melanoma and non-squamous non-small cell lung cancer (NSCLC), and in 2016 atezolizumab (anti-PD-L1 mAb) was greenlighted to treat metastatic NSCLC patients who had progressed after chemotherapy [94].

**Fig. 3:**
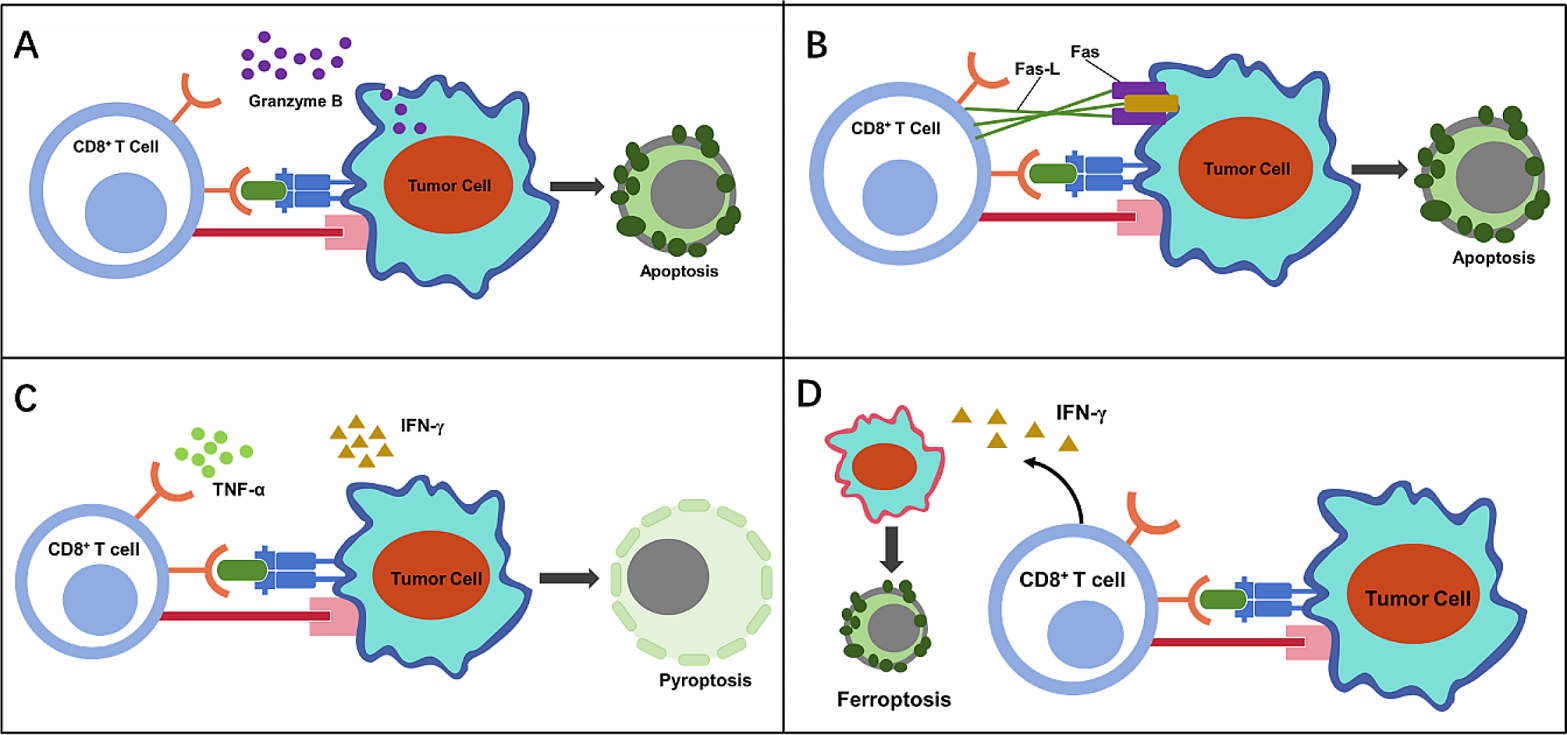
The immunogenic cell death induced by activated CD8^+^ T cells. (**A**) CD8^+ ^T cells induce apoptosis of tumor cells through granzyme B release. (**B**) CD8^+^ T cells induce apoptosis of tumor cells through death receptor ligand. CD8^+^ T cells induce (**C**) pyroptosis (**D**) or ferroptosis of tumor cells by secreting cytokines

CD8^+^ T cell phenotype is a key player in anti-tumor immunity, orchestrating immunogenic cell death in cancers through several mechanisms (Fig. 3). Firstly, immunotherapy-activated CD8^+^ T cells trigger apoptotic cell death via release of perforin-granzyme or through Fas-Fas-L interaction. Upon encountering, CD8^+^ T cells perforate tumor cell membranes and unleash granzyme B into the cytoplasm, or alternatively Fas-expressed tumor cells are susceptible to be eliminated by Fas-L-enriched T cells, both initiating apoptosis [19]. Secondly, non-apoptotic cell death can be induced, directly or indirectly, by cytokine-secreting CD8^+^ T cells. Recent findings revealed that in head and neck squamous cell carcinoma blocking CTLA-4 can activate CD8^+^ T cells, releasing cytokines such as IFN-γ and TNF-α in the TME, further activating gasdermin intracellularly to induce pyroptosis, a programmed lytic cell death in tumors (Fig. 3) [95]. In parallel, IFN-γ released from activated CD8^+^ T cells during immunotherapy may suppress glutamate-cystine antiporter genes, so impairing cystine uptake by tumors. This impairment further promotes lipid peroxidation and accelerates ferroptosis in tumor cells [22].

**Fig. 4:**
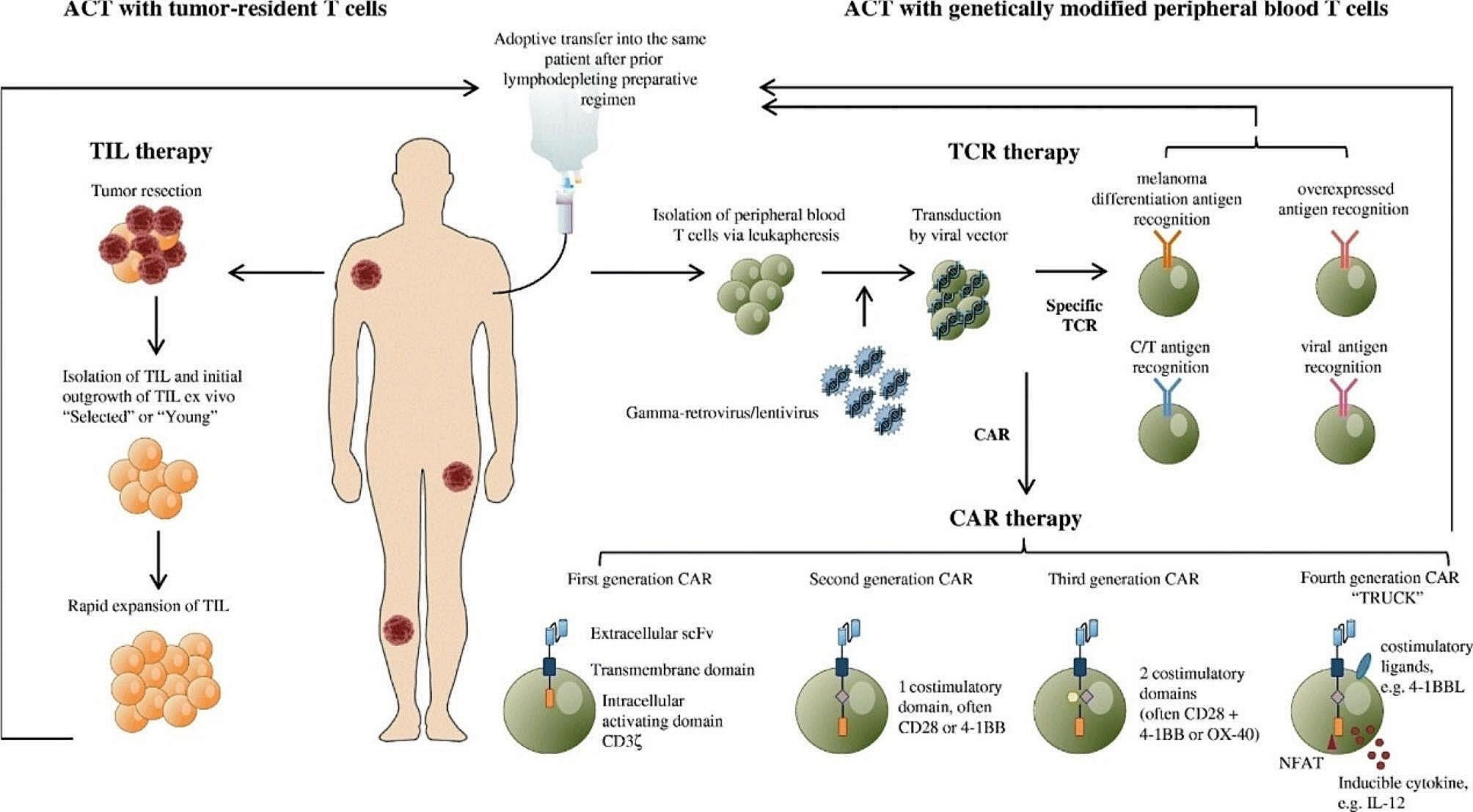
Schematic representation of different adoptive T cell therapy modalities. In TIL-ACT therapy, tumor-resident T cells are isolated and rapidly expanded in vitro after surgery or biopsy. The same patient receives lymphodepletion, followed by infusion of expanded T cells back to the patient. In ACT with genetically modified T cells, T cells in the peripheral blood of patient are isolated and transduced by viral vectors to express specific TCR or CAR for next infusion of modified T cells into the patient. Reproduced with permission [157]

Despite groundbreaking success of ICIs, their overall response rates (ORRs) in patients remain low for most cancer types. Differences in ICI responses among individuals may come from various interplays between aberrant tumors and changing immune cells, where significant increases in immune cell populations, such as CD8^+^ T cells, after ICI treatment can be commonly observed [90, 96]. Accordingly, two major therapeutic strategies are proposed to improve the therapeutic responses in the clinical settings.

Firstly, identification of specific biomarkers in cancers may help predict the patient outcomes when treated using ICIs. Examining CD14^+^CD16b^−^HLA-DR^hi^ in peripheral blood can increase the screening frequency of PD-1 mAb-sensitive stage IV melanoma patient [97]. Lately, breast cancer susceptibility gene 2 was found to be positively related to the therapeutic effect of PD-1 mAb [98]. Similarly, SMARCA4 mutations in patients with NSCLC were associated with improved survival after ICI therapy, suggesting that SMARCA4 detection may help assess the sensitivity of patients to immunotherapy [99]. Thus, timely and precise examination of predictive biomarkers in cancers may obviate non-responsive treatment of ICIs to some extent.

Secondly, synergistic combination of different ICIs or other therapeutics may increase the patient’s response rate and improve therapeutic benefits [100]. A Phase I clinical trial was conducted, where APX005M (anti-CD40 mAb), gemcitabine (DNA synthesis inhibitor) and nab-paclitaxel combined with nivolumab (anti-PD-1 mAb) were administered to treat patients with pancreatic cancer, demonstrating improved results [101]. Moreover, synergistic administration of atezolizumab (anti-PD-L1 mAb) and bevacizumab (anti-VEGF mAb) in treating unresectable hepatocellular carcinoma, currently approved by FDA, showed better tumor response than single administration, although mild-to-moderate adverse reactions were reported [102]. A Phase III clinical trial of atezolizumab combined with bevacizumab and paclitaxel in the treatment of NSCLC also demonstrated that compared with patients given bevacizumab and paclitaxel, the atezolizumab addition improved the survival rate of NSCLC patients [103]. Therefore, optimized combination of different therapy regimens may treat cancers more effectively, depending on enhanced understanding towards more results from real-world evidence studies.

### Neoantigen vaccination

The tumor-specific vaccination that adopts cancerous antigens to activate the host immune system and so produce amplified and long-lasting anti-tumor responses, has become one powerful approach to prevent or suppress tumors [104]. Preventive vaccine lowers the risks of tumor occurrence by protecting the recipients from oncogenic factors due to viral infections, such as human papillomavirus vaccine, while therapeutic vaccine boosts patients’ immune responses and provokes memory immune cells to achieve long-term tumor remission [105]. Research on tumor vaccines focuses on tumor-associated antigens (TAAs) and tumor-specific antigens (TSAs). TAAs refer to molecules of 10-1000-fold increase in tumor cells compared to those in normal cells, such as HER2, p53, and MART-1, while TSAs are neoantigens such as NY-ESO-1 and CEA that are absent or restrictively expressed in normal cells, thus being preferred targets for tumor vaccines [106]. Neoantigens are mutated self-antigens in tumor cells, usually prioritized by whole-exome sequencing and RNA-seq of tumor samples from patients. But a shortage of targetable neoantigens in cancers set back the wide application of tumor vaccination and identified neoantigens induced low intratumoral T cell response, mostly due to heterogeneity in tumor burdens and immunosuppressive TME [107].

To solve those problems, personalized neoantigen vaccines were designed and applied to enhance immunotherapy. With multiple antigen epitopes for patients with glioblastoma, neoantigen-specific CD4^+^ and CD8^+^ T cells from the peripheral blood could infiltrate into intracranial glioblastoma in patients, paving a new avenue for immunotherapy targeting glioblastoma TME [108]. Similarly, peripheral blood mononuclear cells (PBMCs) were collected from individual patients with advanced lung cancers to derive DCs, which were pulsed by neoantigen peptides to obtain autologous DC vaccines for the personalized treatment. As a result, CD8^+^ T cells from PBMCs after vaccination had significantly higher secretion of IL-12 and stronger responses to mutant neoantigens than before vaccination [109]. Moreover, an acidity-responsive nanovaccine containing therapeutic reagents in the core and a model antigen on the surface was developed to greatly improve the antigen presentation by DCs and enhance drug delivery to tumors, shifting the immunosuppressive TME into a milieu in favor of antigen-specific CD4^+^ and CD8^+^ T cells [110].

Neoantigen vaccine may not stand alone to effectively diminish malignant tumors. Its combinations with other therapeutics, such as immune-enhanced adjuvants of granulocyte-macrophage colony-stimulating factor (GM-CSF) or ICIs, can enhance their anticancer effects on solid tumors [111]. For instance, a triple therapy combining neoantigen vaccine that induces the accumulation of CD8^+^ T cells, anti-PD-1 that suppresses immune tolerance signals, and agonist antibody against OX40 that induces T cell memory, was invented to treat mice bearing pancreatic adenocarcinoma of low immunogenicity and poor T cell infiltration, where neoantigen vaccine significantly increased tumor responsiveness [112]. Also, neoantigen DNA vaccination together with anti-PD-1 antibody mediated the colon cancer regression in a CD8^+^ T cell-dependent manner, as opposed to anti-PD-1 antibody alone that failed in tumor elimination [113]. Synthesized polymeric vesicles at nano scale co-delivered peptide antigens and stimulator of interferon genes (STING) agonists to promote DC maturation, eliciting inflammatory cytokine production, costimulatory marker expression, and antigen cross-presentation, leading to mobilization and activation of tumor-specific CD8^+^ T cells. This in return resulted in remarkable improvement in the response to anti-PD-1 and anti-CTLA-4 treatment in murine colorectal adenocarcinoma and melanoma models, respectively [114]. Thereby, personalized cancer vaccines, particularly in rational combination with ICIs, may represent a safe and efficient approach in various malignancies.

### Cellular immunotherapy

Adoptive cell therapy (ACT) ushers cancer treatment into a new era and becomes a milestone in personalized medicine. T cell-based ACT depicts that a number of T cells are isolated from patients with various cancers and induced to transform and proliferate ex vivo, before these therapeutic cells gaining specificity for tumor cells are infused back to patients to improve tumor killing (Fig. 4) [115, 116]. Initially, the therapeutic cells used in ACT were sourced from the patient’s own TILs, but this method is limited by the low abundance of TILs and the inability to efficiently improve tumor dismantling [117]. Alternatively, T cells sourced from the peripheral blood of the patients are genetically modified with surface receptors that recognize tumor-specific antigens, cytokines, or signal transduction molecules [118].

Among them, CAR-T and TCR-T are the most developed immunotherapeutic strategies in the clinical settings. CAR-T cells express chimeric antigen receptor (CAR) molecules on the surface of T cells through gene editing. The process of recognizing tumors by CAR-T cells rely on the unique components of CAR rather than MHC molecule, so CAR-T cells can overcome the immune escape caused by the loss of MHC molecules to some extent [119]. Also genetically modified, TCR-T cells express tumor antigen-specific TCR on the surface of T cells. TCR-T cells can recognize a broad spectrum of antigen peptides presented by MHC molecules, including tumor cell surface antigens, intracellular antigens and neoantigens resulting from tumor mutations [120]. Given the fact that most proteins are expressed intracellularly and CAR-T cells only identify cell surface antigens, TCR-T thereby has a wider tumor antigen selectivity than CAR-T, whereas CAR-T cells are superior to TCR-T cells in terms of target affinity and therapeutic dosage [121]. Both regimens have shown remarkable effectiveness in treating hematological malignancies [122].

In contrast, ACT has achieved less success in treating solid tumors, commonly due to inadequate tumor infiltration and low T cell functionality and persistence [123]. The highly suppressive TME and antigen diversity in solid tumors often invalidate adoptive T cells and promote tumor immune escape, worsened by T cell exhaustion and off-target side effect, thwarting the therapeutic benefits. For CAR-T therapy, antigenic heterogeneity and antigen loss during treatment are two major problems, which may be simultaneously tackled by the strategies to target one surface-expressed antigen using CAR T-cells while also triggering endogenous T-cell responses against additional tumor antigens. In a recent study, vaccine-enhanced CAR-T cells effectively produced IFN-γ, improving the anti-cancer activity, and actively recruited and activated DCs in the tumors with simultaneous IL-12 secretion, triggering the antigen spreading to prevent antigen-negative tumor escape [124]. Through this enhancement, the endogenous immune system was primed, where robust CD4^+^ and CD8^+^ T-cell responses were gained against non-CAR tumor antigen, greatly increasing the tumor infiltration.

To strengthen CAR-T cell functionality and persistence, activation of TGF-β by repetitive challenges can epigenetically reprogram T cells toward a stem-like memory state and promote the robust expansion of human tissue-resident memory CD8^+^ CAR-T cells (CAR-T_RM_), attracting CAR-T cells to accumulate and eventually eliminate solid tumors [125]. Metabolic strategies were carried out to support T_RM_ differentiation and durable function, and to facilitate tissue residency of memory CD8^+^ T cells in solid tumors for TIL enrichment, therefore maintaining their effector functions for improved prognosis [126]. However, it needs to be clarified that the phenotypic mixture of CD4^+^ and CD8^+^ T cells in CAR-T preparation has been long noted. As we here focus the role of CD8^+^ T cells in cancer immunotherapy, the anti-cancer efficacies of CD4^+^ T cells are by no means excluded. Similarly, CD4^+^ T cells can actively respond to mutant antigens, so mediating the tumor elimination [127]. In fact, patients with chronic lymphocytic leukemia, who were infused using CAR-T cells a decade ago and achieved complete cancer remission, exhibited highly activated CD4^+^ T cells that dominated the CAR-T cell population, corroborating a long-persisting CD4^+^ T cells [128].

As Claudin 18.2 (CLDN 18.2) is highly expressed in gastric cancers, humanized anti-CLDN18.2 antibodies were synthesized with specific binding affinity, and CLDN 18.2-expressing CAR-T cells were intravenously injected into mice bearing subcutaneous xenografts of gastric tumors. Consequently, elevated amounts of TNF-α, IL-2, and IFN-γ were produced by CAR-T cells treatment only in CLDN18.2-positive cancer cells, where predominant CD8^+^ T cells were evidenced, corroborating a persistent and highly tumor-infiltrating CAR-T therapy [129]. In this study on-target off-tumor toxicity as an adverse effect was not observed. In 2022, US FDA approved the investigational new drug application of CLDN 18.2-targeted CAR-T therapy in a Phase 1 clinical trial for the treatment of adult patients with relapsed or refractory gastric or pancreatic cancers, where insofar an acceptable safety profile was reported along with a mild cytokine release syndrome occurred in the majority of patients [130]. Two patients with metastatic pancreatic cancer who received CLDN 18.2 CAR-T cell therapy showed significantly increased amount of CD8^+^ T and Treg cells in peripheral blood, leading to well controlled tumor progression and reduced lung metastases [131]. Of note, heightened peak value and augmented copy number of CLDN 18.2 CAR were documented, representing successful expansion and persistence of functional CAR-T cells [130, 131].

For TCR-T cell therapy, most challenges come from off-tumor toxicity and tumor resistance. Off-tumor toxicity is generated by either on- or off-target detrimental effects, where on-target off-tumor toxicity is associated with antigen expression in normal tissues aside from those in cancers, and off-target off-tumor toxicity is related to the cross-reactivity of TCR by recognizing other antigens than designated one in normal cells [120]. These toxicities caused severe morbidity and even death in patients. The tumor-targeting specificity and precision of TCR can be improved by high-throughput screening and bioinformatics analysis to select the TCR of optimal affinity and avidity. Tumor resistance can be primary or secondary, whereas primary resistance mainly results from the antigen heterogeneity in tumors, and secondary resistance is acquired after TCR-T therapy onsets mostly due to the loss or attrition of MHC-I or upregulated immune checkpoints on tumor cells [132]. For instance, the interaction between TCR and PD-1 had a negative regulatory role in T cell antigen recognition [133, 134]. Thus, combined administration of genetically engineered T cells and ICIs may cope with those challenges to enhance the therapeutic efficacy of infused T cells [135].

Without genetic modification, ACT with TILs (TIL-ACT) first extracts the infiltrating lymphocytes from the patients’ tumor tissues through biopsy or surgery, and then expand them in vitro with IL-2 stimulation, followed by infusion of TILs back into the same patients for treatment. With cumulative experiences and technical advances on engineered ACT therapy, understanding towards maintaining T cell function and penetrating TME enables TIL-ACT to be actively applied in clinical studies. As TILs are naturally and originally occurring TME-infiltrated cells, they hold migration privilege back to the same tumor and possess minimal off-target toxicity, recycled by negative TCR selection [136]. Patients with metastatic tumors that previously had decent levels of intratumoral and stromal CD8^+^ TILs in a network with activated myeloid population can benefit from TIL-ACT treatment [137]. Research efforts have been continuously made to promote TILs manufacturing and standardize the therapy regimen to advance the efficacy of TIL-ACT and ensure optimal patient outcomes. Currently, TIL ACT has been gained FDA approval as the first-of-its-kind ACT therapy for solid tumors with other clinical trials ongoing.

### Oncolytic virotherapy

Taking advantage of intrinsic lytic characteristics in naturally occurring viruses, oncolytic virotherapy is a triple interplay among the virus, the host immunity and the TME. Genetically modified oncolytic viruses (OVs) are equipped with exogenous materials to improve the infection specificity to cancer cells, promote the viral replication inside cancers, and ensure the tolerable biosafety, while keeping the normal cells minimally impacted and the tumor cells maximally lysated [138]. Talimogene laherparepvec (T-VEC) is the first approved oncolytic virotherapy for treatment of patients with unresectable or metastatic melanoma in 2015. T-VEC employs herpes simplex virus type 1 (HSV-1) with oncolytic properties but inactivates deleterious endogenous substances, such as neurotoxic factors, and inserts immunologically active boosters (e.g., GM-CSF encoding genes) to enhance further immune responses [139]. Through intratumoral injection, T-VEC disintegrates tumor cells and releases TAAs to provoke local or even distant anti-cancer immunity, recruiting immune cells to reverse TME and elevating CD8^+^ T cell responses. As a result, durable response rate and overall survival of patients with advanced melanoma were superior receiving T-VEC as first-line therapy [140]. Similarly, HSV-1-based Teserpaturev/G47Δ has been approved for the treatment of malignant glioma in Japan, and this therapy currently enters clinical trials of other solid malignancies, including prostate cancer and recurrent olfactory neuroblastoma [141].

Oncolytic virotherapies in combination with other treatments have been experimented in pre-clinical and clinical studies. Vesicular stomatitis virus (VSV), owing to its high sensitivity to type-I IFN inhibition and tropism to type-I IFN-deficient tumors, was overexpressed with IFN-β genes, which induced potent CD8^+^ T cell responses in murine models of subcutaneous B16 tumors and significant reduction of tumor volume. Compared to treatment using ICI alone, combinatorial treatment using VSV-based therapy together with ICI showed better anti-tumor efficacy with higher survival rates [142]. Furthermore, in lymphodepleted mice bearing B16 tumors, VSV-based therapy plus type-I IFN resistant CD8 CAR-T cells provided better tumor inhibition and higher survival rate, than type-I IFN resistant CD8 CAR-T cells alone or VSV plus wide-type CD8 CAR-T cells [143]. Thus, OV combined with ACT therapy improves the abundance of tumor reactive CD8^+^ T cells. Nevertheless, VSV-associated type-I IFN was also found to promote apoptosis in CD8 CAR-T cells where CAR was highly expressed, resulting in negative therapeutic effect [143]. It follows that OV fabrication is a multifactorial process, not automatically beneficial for combination immunotherapy. A recent Phase III study revealed that treatment using T-VEC plus pembrolizumab in immunotherapy-naïve patients with advanced melanoma, did not show improved progression-free survival or overall survival, compared to placebo-pembrolizumab treatment [144].

### Nanomedicine

**Fig. 5:**
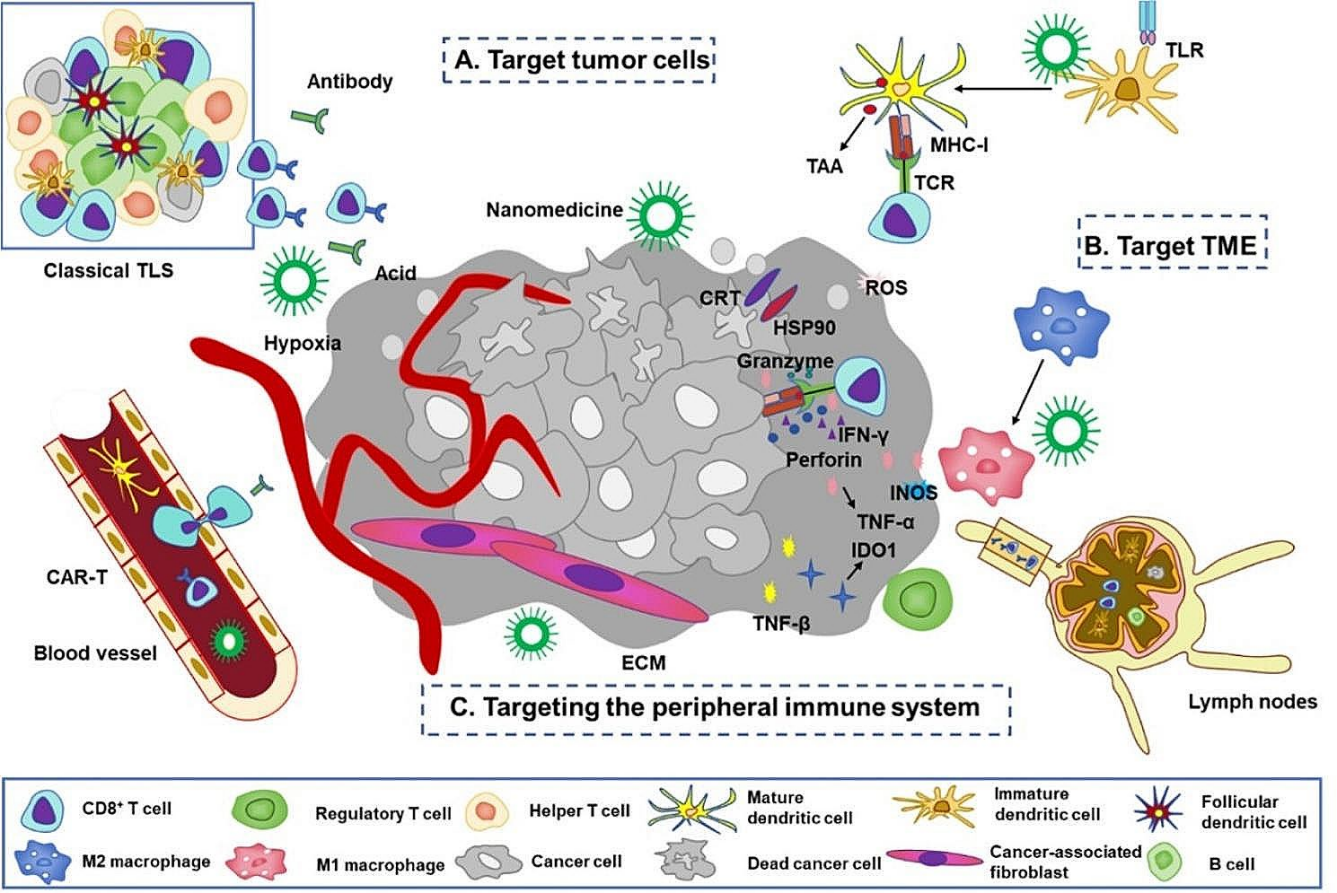
Immunotherapy combined with nanomedicine. (**A**) Target tumor cells and induce immunogenic cell death. Nanomedicine encapsulating photosensitizers can target and enter tumor cells through enhanced permeability and retention effect of tumors, and then release therapeutic cargoes, triggering the translocation of calcium reticulum protein to the tumor cell membrane. The synergistic effect with reactive oxygen species produced by photosensitizers leads to cell death in tumors [147]. CRT, calcium reticulum protein; ROS, reactive oxygen species. (**b**) Target the TME. Nanomedicine coupled TLR agonist can induce DC maturation and prolong the duration of antigen presentation. In addition, the designed nanomedicine can affect the function of TAMs and manipulate TAMs to repolarize into M1 pro-inflammatory phenotype, producing TNF-α and iNOS [151]. iNOS, inducible nitric oxide synthase. (**C**) Target the peripheral immune system. Nanomedicine can encapsulate genes encoding CAR or TCR, which can selectively bind to CD8^+^ T cells in circulating blood, initiating receptor mediated endocytosis to internalize nanomedicine. With the release of mRNA from CD8^+^ T cells, T cells are reprogrammed to express CAR or TCR, inducing anti-tumor responses [152]. ECM, extracellular matrix

In the recent decade the combination of cancer nanomedicine and immunotherapy has attracted much attention. To improve the delivery of immunotherapeutic agents to tumor targets, nanotechnologies are implemented for enhancement of specific local tumor immune responses that are more reliable and durable with less systemic toxicity [145]. Three basic targeting strategies of nano-immunotherapy have been developed as follows (Fig. 5) [146].

Firstly, tumor cells can be targeted to induce immunogenic cell death. A multimodal nanoparticle was constructed with coordination polymers containing Zn^2+^ and phosphate groups of an oxaliplatin prodrug in the core and coated with the photosensitizer pyrolipid conjugate [147]. In bilateral tumor-bearing mice of colon cancer model, intraperitoneal injection of nanoparticles induced an effective synergy between chemotherapy and photodynamic therapy upon light irradiation and activated immune response, triggering calreticulin relocation onto the tumor cell surface that summoned APC to process tumor antigens and activated antigen-specific CD8^+^ T cells. The nanoparticles, utilizing an enhanced permeability and retention effect to highly accumulate inside tumors, provide an immunogenic milieu in TME and a systemic tumor-specific immune response for the ensuing intraperitoneal injection of PD-L1 antibody, amplifying its antitumor efficacy [147]. By changing the photosensitizer moiety to a chemical drug, similar nanoparticles were fabricated and administered in combination of anti-PD-L1 antibody, greatly increasing the intratumoral infiltration of CD8^+^ T cells. As a result, this nanomedicine not only afforded complete tumor eradication, but also prevented tumor formation when mice of tumor remission were challenged again with cancer cells [148]. Therefore, this combination therapy may successfully prompt strong and long-lasting antitumor immunity.

Secondly, immune microenvironment can be targeted to revive tumor specific CD8^+^ T cells and increase their proliferation and infiltration. Nanoparticles based on functional peptide self-assembly and conjugated with Toll-like receptor (TLR7 and TLR8) agonist effectively induced DC maturation and extended the duration of antigen presentation, so potentiating neoantigen-specific stem-like CD8^+^ T cells to optimize the anti-tumor immunity [149]. In a murine lung cancer model, nanosized liposomes were loaded with dual inhibitors against both exosome biogenesis and release and coupled with antibodies against epithelial cell adhesion molecule, targeting and shifting cancer associated fibroblasts into quiescent fibroblasts. This TME reversal increased the cytotoxic T cell infiltration and augmented antitumor efficacy of PD-L1 antibody when administered synergistically [150]. Moreover, magnetic iron oxide nanoparticles with cationic polymer functionalization were internalized by myeloid-derived suppressor cells (MDSCs) (mainly TAMs) in brain tumor. Under radiation the nanoparticles repolarized MDSCs into M1 pro-inflammatory phenotype, producing a large amount of TNF-α and inducible nitric oxide synthase to relieve the suppression of CD8^+^ T cells in the inhibitory TME, which finally enhanced the anti-tumor efficacy [151].

Thirdly, the peripheral immune system can be targeted to reactivate and amplify CD8^+^ T cells. To this end, polymeric nanoparticles with surface conjugation to anti-CD8 antibody were loaded with mRNAs encoding CAR and TCR, which could selectively bind to CD8^+^ T cells in the circulating blood of murine models bearing human leukemia, prostate cancer, and hepatocellular carcinoma, to initiate receptor-mediated endocytosis. Following the mRNA unleashing in CD8^+^ T cells, T cells were reprogrammed to transiently express tumor-specific CAR or virus-specific TCR, which induced tumor regression comparable to therapeutic outcome of ACT [152]. Notably, tertiary lymphoid structure (TLS) is mainly formed by aggregation of immune cells such as CD8^+^ T and CD20^+^ B lymphocytes, which is observed in metastatic solid tumors and correlated to improved patient survival rate [153]. The presence of TLS was found in 94% of patients with high-grade ovarian cancer, and a strong co-occurrence of the infiltrated CD8^+^ T cells and B cell lineages was confirmed [154]. Recently, a nanovaccine made of Epstein-Barr virus nuclear antigen 1 (neoantigen) and a bi-adjuvant of Mn^2+^ (STING agonist for T cell activation) and cytosine-phosphate-guanine (TLR-9 agonist for B cell activation) was formulated and injected into mice bearing nasopharyngeal carcinoma. After administration, TLS was formed and normalized blood and lymph vessels were detected in tumor tissues, correlated with increased presence of CD8^+^ T lymphocytes in tumor and peripheral blood [155]. Thus, targeting TLS to promote the functional maturation of T and B cells and to obviate the adverse reactions can be a new direction to advance integration of nanomedicine and immunotherapy.

## Conclusion

In this review we discussed the basics regarding the life cycle of CD8^+^ T cells, as well as how they develop into frontline warriors with robust anti-cancer activity. Simultaneously, we delved into the mechanism of tumor immune escape with recent research findings and deliberated the immunotherapy strategies in experimental and clinical medicine with new findings and ongoing challenges. Put together, CD8^+^ T cell plays an important role in anti-tumor immunity, as CD8^+^ T cell-based immunotherapies are becoming an indispensable component in the frontline cancer therapy. While continuous efforts towards the clinical applications of CD8^+^ T cell-based therapeutics are doubtlessly necessitated, the fundamental understanding of both tumor and T-cell biology, such as tumor heterogeneity and checkpoint blockade, to better design the immunotherapy regimen and to assess therapeutic outcome, is greatly warranted.

## Data Availability

Not applicable.

## Abbreviations

ACT: adoptive cell therapy
APCs: antigen-presenting cells
CAR-T: chimeric antigen receptor T
CAR-T_RM_: tissue-resident memory CAR-T cells
CTLA-4: cytotoxic T lymphocyte antigen 4
CLDN: 18.2-Claudin 18.2
CXCR3: chemokine receptor CXC motif 3
DCs: dendritic cells
EVs: extracellular vesicles
FDA: Food and Drug Administration
HIF-α: hypoxia-inducible factor-α
HLA: human leukocyte antigen
HSV-1: herpes simplex virus type 1
IDO1: indoleamine 2, 3-dioxygenase 1
IFN-g: interferon g
LAG: 3-lymphocyte activation gene-3
MDSCs: myeloid-derived suppressor cells
MHC: major histocompatibility complex
MHC I: major histocompatibility complex class I
NSCLC: non-small cell lung cancer
ORRs: overall response rates
Ovs: oncolytic viruses
OVV: oncolytic virus vaccine
PD-1: programmed cell death protein-1
PD-L1: programmed cell death-ligand 1
PDAC: pancreatic ductal adenocarcinoma
PBMCs: peripheral blood mononuclear cells
STING: stimulator of interferon genes
TAAs: tumor-associated antigens
TAMs: tumor-associated macrophages
TA-TLS: tumor associated TLS
TCR-T: T-cell receptor T
TGF-b: transforming growth factor b
TIGIT: T cell immunoglobulin and ITIM domain
TILs: tumor-infiltrating lymphocytes
TIM-3: T cell immunoglobulin and mucin-domain containing-3
TLS: tertiary lymphoid structure
TNF-α: tumor necrosis factorα
TME: tumor microenvironment
Tregs: regulatory T cells
TSAs: tumor-specific antigens
T-VEC: talimogene laherparepvec
VEGF: vascular endothelial growth factor
